# Printed Strain Sensors Based on an Intermittent Conductive Pattern Filled with Resistive Ink Droplets

**DOI:** 10.3390/s20154181

**Published:** 2020-07-28

**Authors:** Daniel Zymelka, Takahiro Yamashita, Xiuru Sun, Takeshi Kobayashi

**Affiliations:** National Institute of Advanced Industrial Science and Technology, Tsukuba, Ibaraki 305-8564, Japan; takahiro-yamashita@aist.go.jp (T.Y.); shuujo.son@aist.go.jp (X.S.); takeshi-kobayashi@aist.go.jp (T.K.)

**Keywords:** strain sensor, printed electronics, hybrid sensor structure

## Abstract

In this study, we demonstrate a strain sensor fabricated as a hybrid structure of a conductive intermittent pattern with embedded single droplets of a functional resistive ink. The main feature of our proposed sensor design is that although the intermittent pattern comprises the majority of the entire sensor area, the strain sensitivity depends almost selectively on the resistive droplets. This opens up the possibility for fast and inexpensive evaluation of sensors manufactured from various functional materials. As the use of resistive ink was limited to single droplets deposition, the required ink amount needed to build a sensor can be considerably reduced. This makes the sensors cost-effective and simple for fabrication. In this study, our proposed sensor design was evaluated when a carbon-based ink was used as the resistive material incorporated into an intermittent structure made of silver. The developed strain sensors were tested during bending deformations demonstrating good strain sensitivity (gauge factor: 7.71) and no hysteresis within the investigated strain range.

## 1. Introduction

Strain sensors have a long history of being used in various engineering fields. Aside from aerospace and automotive applications, strain sensors are also widely used to monitor civil infrastructures [1,2]. Conventional metal foil strain sensors typically comprise a copper–nickel alloy, commonly known as constantan. Constantan-based sensors are of particular interest, mainly owing to their low thermal coefficient of resistance. Such sensors are generally fabricated using a photolithography etching process that involves several fabrication steps and materials. Nonetheless, strain sensors have significantly evolved during the last years. Recent progress in additive manufacturing, widely used for flexible printed electronics [3], opens up new possibilities for the cost-effective fabrication of sensors using diverse materials. As printable constantan-based inks are not commercially available, printed strain sensors are generally manufactured from other strain-sensitive materials based on graphite [4], silver [5], PEDOT:PSS [6,7,8], graphene [9], carbon nanotubes (CNT) [10] or composites of these materials [11,12,13,14]. With regard to applications, most current studies focus on the use of strain sensors for wearable devices and human motion detection [15,16,17]. Nonetheless, other original applications of printed sensors have been demonstrated for strain monitoring of hip prosthesis [18], handguns [19], and tires [20]. Moreover, printed sensors have been successfully used to monitor vital signs [21]. Other interesting applications include structural health monitoring of aircrafts [22] and civil infrastructure [23,24], where large-area sensors and arrays of multiple strain sensors are desirable.

Besides the materials used, strain sensors may also be differentiated in terms of their shape. Depending on applications, instead of the most commonly used linear sensing grid, strain sensors can take a form of other diverse geometries. For instance, in our previous studies, we demonstrated an asterisks-shaped strain sensor capable of performing omnidirectional strain analysis [25]. In contrast to the conventional sensors (with linear sensing grid), the demonstrated omnidirectional strain sensor exhibit almost uniform sensitivity at various installation angles. Another recently demonstrated the alternative concept of strain sensors consists of repetitive patterns of the overlapping rings and diamond-shaped electrodes [26]. The authors have shown that such specific geometries made of silver nanowires exhibit high stretchability and good strain sensitivity. Other very interesting groups of sensors consist of fiber-shaped sensors [27,28] and crack-based sensors [29,30,31]. Especially the crack-based strain sensors have recently attracted a lot of attention. In this type of sensors, the crack patterns are spontaneously generated when tensile stress is applied to such structures. The mechanical deformations at the crack induce variations in the electrical resistance due to changes in the crack opening. It makes these sensors very sensitive to external mechanical deformations.

All above-mentioned research shows great promise for future practical applications. However, although the previously reported printed strain sensors differ in terms of materials used and shapes associated with their specific target applications, all these sensors have one common feature. They are entirely constructed using a strain-sensitive material that defines both the sensor shape and sensing properties. In this study, we evaluate an alternative concept for a strain sensor whose design is based on a hybrid construction of a conductive intermittent pattern with embedded single droplets of resistive functional ink, i.e., an ink used to build electrically resistive elements in the sensor structure providing strain sensitivity. The principle of operation of the sensor is similar to the conventional sensors and is based on monitoring of the electrical resistance changes in the entire sensor structure subjected to mechanical deformations. However, for the proposed sensor design, the electrical resistance of the intermittent conductive pattern was much lower than the resistance of resistive elements. We demonstrate that owing to such a configuration, the sensitivity of the entire sensor depends almost selectively on the properties of the resistive elements. Although the intermittent pattern made of silver comprises over 90% of the entire sensor, its contribution to the measured resistance change was only approximately 1%. This is the key feature of the developed strain sensor. The resistive functional inks define sensor sensitivity while the intermittent conductive patterns specify the sensor shape or, in other words, a path along which strain is measured in the points corresponding to the positions specified by the resistive elements. As the use of functional ink was limited to the deposition of single droplets, the ink volume required to fabricate a sensor can be significantly reduced. Such a fabrication process may be particularly promising for composite materials based on CNT or graphene that exhibits good mechanical properties and are yet relatively expensive. Moreover, owing to such a fabrication approach, the ink rheology does not need to be strictly controlled in terms of printability. This opens up the possibility for a fast and inexpensive evaluation of sensors made of various functional materials. Another important advantage of such fabrication is the possibility of designing strain sensors with a desirable electrical resistance.

In this study, the proposed concept for the sensor design was evaluated when carbon-based ink droplets were incorporated into an intermittent pattern made of a screen-printed silver ink. In terms of mechanical properties, the developed sensor exhibits good strain sensitivity and no hysteresis within the investigated strain range. The demonstrated concept makes the sensors cost-effective and simple for fabrication. Their size, shape, and number of intermittent segments can be redesigned depending on desired future applications.

## 2. Experimental Methods

### 2.1. Construction of the Strain Sensor

**Figure 1:**
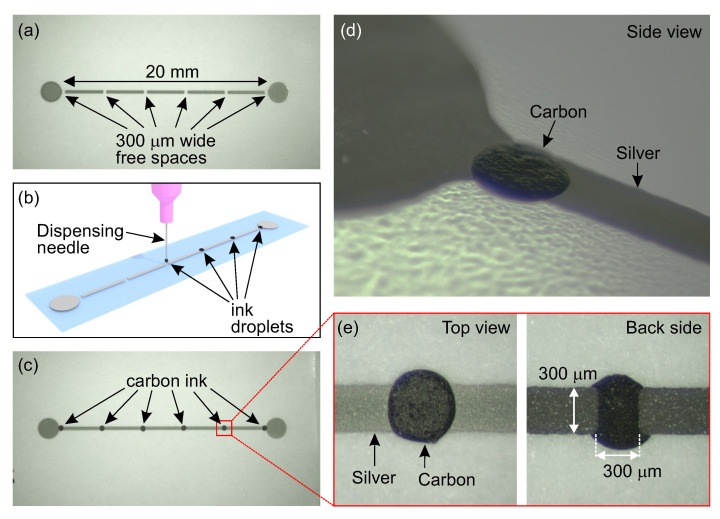
(**a**) Printed pattern of the intermittent structure made of silver ink. (**b**) Deposition of single droplets of the resistive carbon ink. (**c**) Top view on the fabricated strain sensor. (**d**) Side view on a carbon droplet. (**e**) Zoom in dried droplets made of the resistive carbon ink.

The preparation of the strain sensors begins from the printing of the intermittent conductive pattern demonstrated in Figure 1a. Silver ink (Genes’ink CS21306, France) was screen-printed onto 20 μm-thick polyethylene terephthalate (PET) substrate. A stainless steel mesh (Asada Mesh HS-D 650/14, Japan) was used for high-resolution printing of 20.3-mm-long and 0.3-mm-wide intermittent pattern, divided into five segments, each 3.7-mm long. The gap between the two segments was 0.3 mm. After the printing, such patterns were cured in a conventional convection oven at 130 ${}^{\circ }$C for 30 min. The average electrical resistance of each segment was 1.65 ± 0.02 Ω. In the next step, the free spaces of the intermittent structure were filled with carbon-based ink (TOYOBO DY-200L-2, Japan) (Figure 1b). The carbon ink had a relatively high concentration of solid content of 45 wt.% and volume resistivity of 1.0 × 10${}^{-1}\;\Omega \;\cdot$ cm. For this study, the carbon ink was selected owing to its high resistivity, which is desirable with regard to the demonstrated sensor design. According to the proposed concept, the resistance of the sensing elements should be higher than the conductive intermittent pattern (Section 3.3). The functional ink was used in a very small quantity through a single droplet deposition using a dispenser (Musashi Engineering ML-5000XII, Japan). The dispenser had adjustable pressure and discharge time. The pressure of 0.35 MPa and discharge time of 70 ms were set to form droplets sufficiently large to fill the 0.3 mm × 0.3 mm free spaces in the intermittent pattern and to provide electrical contact between the consecutive silver segments (Figure 1c). After carbon ink deposition, the entire sensor structure was dried in a convection oven at 130 ${}^{\circ }$C for 30 min. The electrical resistance of dried single carbon droplets was 32.6 ± 2.3 Ω, while the entire hybrid (silver–carbon) structure had a resistance of 199.3 ± 8.4 Ω. For carbon ink deposition, a dispenser that enables precise control in terms of the desired droplets size was used; however, the alignment for the ink deposition was manual. A syringe with ink and needle was held in a hand. The accuracy of resistive ink deposition can be improved if a dispenser with an automated arm is used. Alternatively, other methods such as inkjet printing or microplotter can be implemented. However, in this study, the dispenser was selected because it enables deposition of inks with a broad range of viscosities, i.e., the ink printability does not have to be strictly controlled. Figure 1d,e shows single droplets after the curing process, incorporated into the intermittent silver pattern.

### 2.2. Electrical Configuration of the Sensor

**Figure 2:**
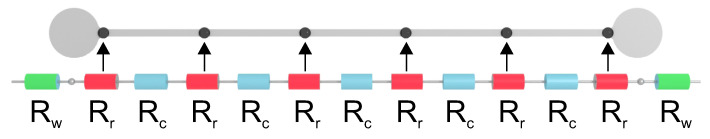
Electrical configuration of the developed strain sensor. $R_{c}$ is the electrical resistance of single segments of the intermittent structure, $R_{r}$ resistance of a single resistive element, n is a number of the resistive elements in the entire sensor structure, $R_{w}$ resistance of wires attached to the sensor.

The proposed hybrid construction for the strain sensor differs from that for the standard sensors. Instead of one resistive sensor structure, several small resistive elements (made of carbon) were connected in series within the intermittent pattern made of a good electrical conductor (silver). The electrical configuration of such a sensor is illustrated in Figure 2. The electrical resistance of the sensor can be described by the Equation (1), where $R_{r}$ is the resistance of a single resistive element, $R_{c}$ is the resistance of single silver segments of the intermittent structure, n is the number of the resistive elements embedded in the entire sensor, $R_{x}$ contact resistance between $R_{r}$ and $R_{c}$.

(1) $$R_{total}=R_{r}\cdot n+R_{c}\left(n-1\right)+R_{x}\cdot 2n$$

### 2.3. Experimental Setup and Data Acquisition System for Strain Analysis

**Figure 3:**
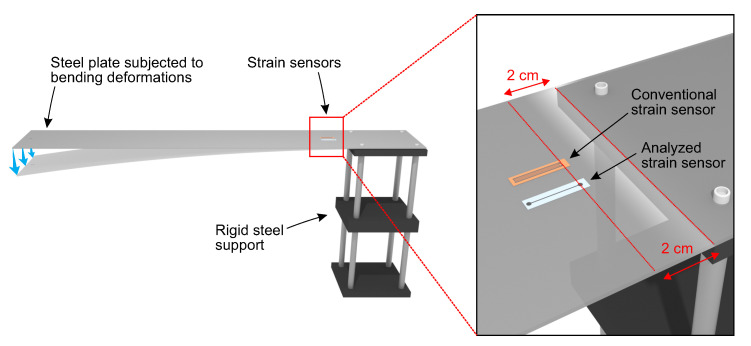
Experimental setup used to calibrate the investigated strain sensors.

**Figure 4:**
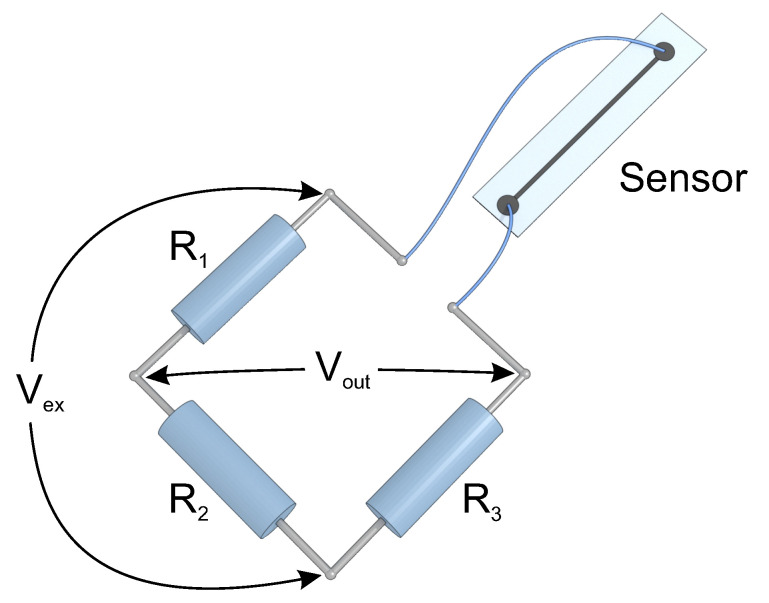
Schematic of a strain sensor connected to the quarter Wheatstone bridge circuit.

The developed strain sensors were bonded to a steel plate as demonstrated in Figure 3. The plate was 70-cm long, 12-cm wide, and 2-mm thick and was installed on a rigid support. Sensors were attached to the plate using a cyanoacrylate adhesive (Loctite 414, Henkel, Germany). Subsequently, the electrical wires were bonded to the round electrodes of sensors using a silver-filled epoxy adhesive (M.G. Chemicals 8331, Canada). The sensors were connected to the quarter Wheatstone bridge circuit (Figure 4) with the excitation voltage of 2.4 V delivered from a power supply (Keysight E36311A, USA). The output voltage was measured using a 24-bit analog input module (NI 9238, USA) connected to a chassis (NI cDAQ-9188, USA). The measurements were registered and analyzed using a specially prepared computer program (NI LabView, USA).

### 2.4. Calibration

The electrical resistance of sensors attached to the steel plate varies with the degree of axial bending. To determine the sensitivity of these sensors, the relative change in resistance ($\frac{\Delta R}{R_{0}}$) was measured as a function of mechanical strain (ε). The sensitivity of a strain sensor is defined using the gauge factor (GF), which is expressed as follows:

(2) $$GF=\frac{\Delta R/R_{0}}{\varepsilon }$$

In this study, the conventional strain gauges (Kyowa KFGS-20-120-C1-11, Japan) were used to provide reference strain measurements and to calibrate the developed sensors. The reference strain sensors were installed on the plate aside to the investigated sensors (Figure 3).

### 2.5. Characterization

Thickness profiles of the silver line and carbon droplets were measured by using a mechanical profilometer (Surfcorder ET4000M, Kosaka Laboratory Ltd., Japan). Images were taken using an optical microscope (Vision Engineering Lynx EVO, UK). Sensors subjected to various temperatures were characterized inside an environmental test chamber (Espec SH-642, Japan). During the analysis, output signal form sensors was recorded using the same data acquisition system as for strain sensitivity analysis. Temperature was measured using a platinum resistance temperature detector (RTD).

## 3. Results and Discussion

### 3.1. Thickness Profile Analysis

**Figure 5:**
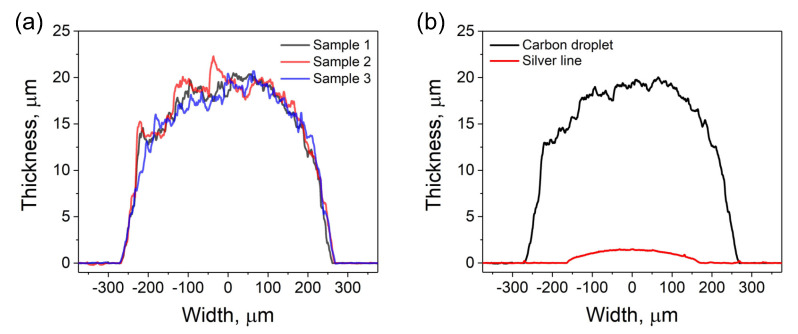
Thickness profile analysis using a mechanical profilometer. (**a**) Comparative analysis of three dried droplets of the carbon ink. (**b**) Average profiles showing the difference between the dimensions of carbon droplets and silver lines.

The analysis of thickness profiles was intended to verify and compare the size of the printed silver lines and resistive elements made of carbon ink droplets. As the resistive elements define the sensor proprieties, at first, the reproducibility of dried droplets size was analyzed. Three droplets were measured across the diameter. The comparative analysis is demonstrated in Figure 5a. The results show that the thickness profiles of all droplets were similar. This is possibly owing to the use of the controllable dispenser that provides the reproducible process for the ink deposition. Subsequently, from the measured data, an average maximal thickness of the carbon layer was calculated and compared to an average maximal thickness of the silver pattern (Figure 5b). The measured thickness taken in the middle of the resistive carbon layer was 19.45 ± 0.57 μm. This value was larger than the thickness of the silver pattern of approximately 1.41 ± 0.05 μm, measured in the same manner. The higher thickness for the resistive elements was mostly related to the difference in the deposition method and relatively high concentration of the carbon ink (45 wt.%). While the silver ink was screen-printed, carbon ink was deposited using the dispenser. Because the electrical resistance of the entire sensor depends on the thickness of carbon layer, i.e., the resistive elements, their controllable deposition may be important for the fabrication of sensors with the desired resistance. By using the dispenser, such a process becomes relatively simple. The thickness of resistive elements can be controlled by the discharge time (the longer discharge time, the more material is deposed), or by changing the concentration of solid content in the functional ink.

### 3.2. Basic Analysis of Strain Sensitivity

**Figure 6:**
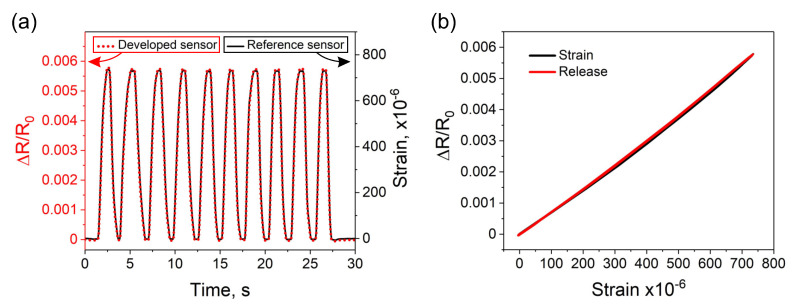
(**a**) Analysis of the developed strain sensor with the hybrid construction under cyclic deformations. The measured output signal from the developed sensor was compared to strain levels measured by the reference conventional strain sensor. (**b**) Analysis of the sensor with the hybrid construction intended to evaluate the linearity of the measured output signal and the presence of hysteresis. The plot shows 10 strain cycles.

**Figure 7:**
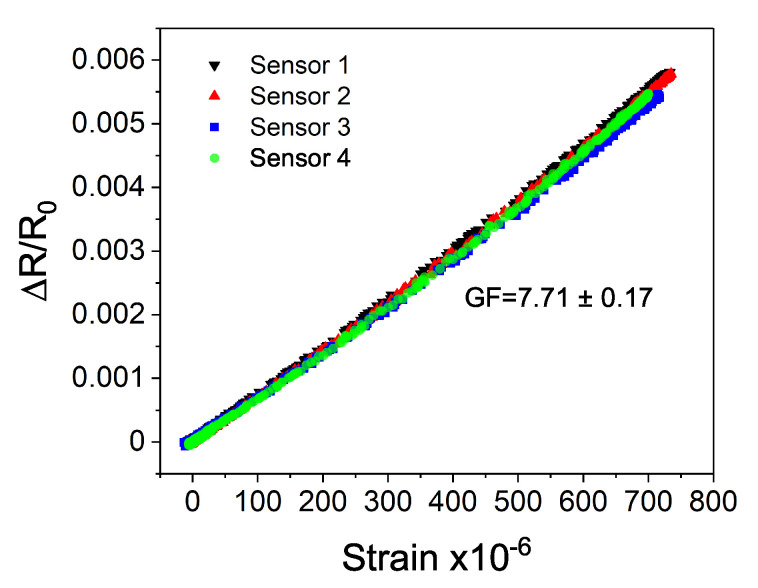
Comparative analysis and calibration of four of the same strain sensors with the hybrid construction. The demonstrated results show good repeatability between the sensors.

The strain sensitivity was analyzed by measuring the variations in electrical resistance of the entire sensor structure when the steel plate with the attached sensors was subjected to mechanical deformations. During the analysis, 10 bending cycles up to approximately 700 microstrain (με) were implemented. While the relative change in resistance was recorded by the developed sensors, the reference strain measurement was simultaneously registered by the conventional strain gauge (Figure 6a). The collected results on the resistance changes were then analyzed as a function of the applied strain, measured by the reference sensor (Figure 6b). Such analysis was intended to evaluate the linearity of the measured output signal. The results show that within the investigated strain range, with the increase of the applied strain and while returning to the initial position, the sensor exhibits a linear output signal and no hysteresis. Next, to calculate strain sensitivity and to assess the repeatability, three additional sensors were fabricated and analyzed under the same conditions. Figure 7 demonstrates a comparative analysis of four of the same sensors. The demonstrated results show good repeatability between the sensors. Within the investigated strain range, all sensors reveal similar linear output signal and no hysteresis. The sensitivity of sensors was calculated based on the Equation (2). The calculated average GF was 7.71 ± 0.17; this higher than the GF of around 2 for typical conventional strain gauges.

**Figure 8:**
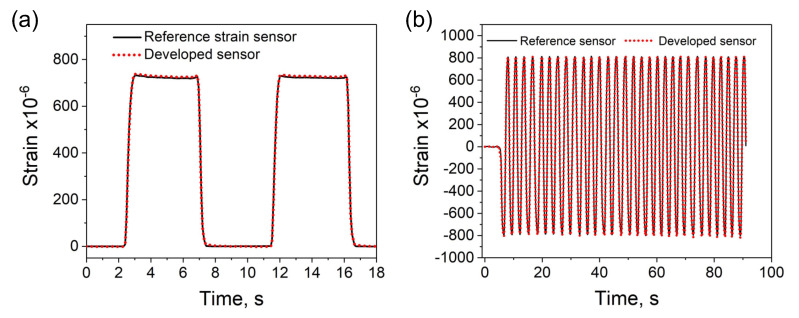
Analysis of the developed sensor under (**a**) static (**b**) and dynamic strains. The recorded output signal shows a good correlation with the reference strain measurement.

Besides the dynamic strain analysis, the sensor was tested under static strains as demonstrated in Figure 8a. Under static bending deformation, at the high state, the output signal remains constant and it goes backs to the low state without a visible delay. At this stage of the analysis, the developed sensor was already calibrated (Figure 7), i.e., the measured output signal, instead of the change in resistance shows measured strains. Thus, to assess the accuracy of the developed sensor, the strain measured by both types of sensors was compared at the high state. The results showed that while the strain indication by the reference sensor was 722.1 ± 1.5 με, strain measured by the developed sensor was 727.9 ± 1.9 με. Thus, both sensors showed similar changes in strain and similar strain resolution. The difference between measured strain values was only about 6 με, indicating good accuracy of the developed sensor within the analyzed strain range. In this study, the analyzed sensors were bonded to a steel plate as demonstrated in Figure 3. Such installation method provides stable strain measurements, but the measuring range was limited to the bendability of the plate. In the present configuration the maximal strain was about 800 με which was sufficient to assess the performance of the sensor. Nonetheless, to provide more information on the strain range, an additional analysis under dynamic bending deformations in both the directions (positive and negative, alternately) was performed (Figure 8b). The collected results show good response time of the developed sensor to the applied external deformation. The strain measurements were consistent with those measured by the reference strain sensor. The above results demonstrates the potential suitability of the developed sensor for both static and dynamic strain analyses.

### 3.3. Discussion and Additional Analysis Concerning Strain Sensitivity

As demonstrated in Figure 2, the hybrid construction of the developed strain sensor differs from the standard sensors that are entirely fabricated from resistive materials. In this study, small carbon-based resistive elements were connected in series within the intermittent pattern made of silver. Nonetheless, because the silver intermittent pattern comprises over 90% of the sensor area, assessing its contribution to strain sensitivity of the entire hybrid sensor structure was crucial. Moreover, assessing the contribution related to contact resistances between $R_{r}$ and $R_{c}$ was necessary.

**Figure 9:**
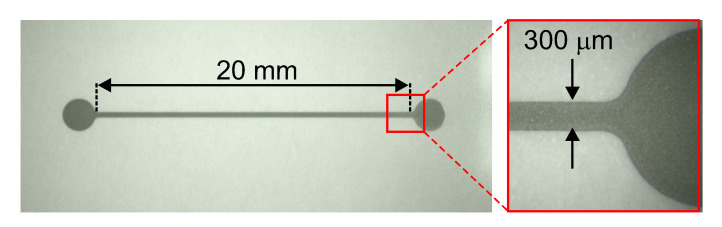
Screen-printed strain sensor entirely made of silver ink.

Initially, a sensor with no resistive elements, entirely made of silver was fabricated using screen printing (Figure 9). It had the same dimensions as the intermittent pattern and was prepared using the same materials and methods. The electrical resistance of such a sensor in the form of a continuous line was 7.79 ± 0.06 Ω. The sensor was calibrated in the same manner as the one with the hybrid structure. The calculated GF was 2.02, which is almost four times less than the GF of the sensor with the hybrid design (GF = 7.71) and almost the same as the GF of commercially available strain sensors.

Nonetheless, the contribution of the silver pattern to the sensitivity of the entire hybrid sensor cannot be assessed based on a simple comparison of GFs. According to Equation (2), the measured resistance change in a sensor subjected to a given strain depends on the GF of the sensor and its electrical resistance. The higher the resistance of a sensor is, the higher will the resistance change be measured. Because the electrical resistance of the silver pattern (7.79 Ω) was lower than the resistance of the entire hybrid sensor incorporating the resistive elements (199.3 Ω) the impact of the silver structure on the strain sensitivity of the sensor was negligible. To illustrate this, we can substitute into Equation (2) the measured GFs, resistances, and strain range of 700 με. For the sensor entirely made of silver (Equation (3)), the estimated resistance change was approximately 0.011 Ω, which is almost a hundred times less than the calculated resistance change of 1.076 Ω for the sensor incorporating the resistive elements (Equation (4)). Although the intermittent pattern made of silver comprises over 90% of the entire sensor (Figure 1c), its contribution to the measured resistance change was only approximately 1%. Because the electrical resistance of carbon layer was higher than the resistance of the silver pattern, the sensitivity of the entire sensor structure depended mainly on the properties of the resistive elements fabricated from functional carbon ink. This is the key feature of the developed strain sensor. It enables combining various resistive functional inks that define the sensor sensitivity with the intermittent conductive structure that specifies the sensor’s shape. Moreover, the ink amount needed to build a sensor can be significantly reduced. Such a fabrication process may be especially interesting for materials like CNT or graphene that exhibits good mechanical properties and are yet relatively expensive.

(3) $$\Delta R_{silver}=\varepsilon \times GF_{silver}\times R_{silver}=700\times 10^{-6}\times 2.02\times 7.79\;\Omega =0.011\;\Omega$$

(4) $$\Delta R_{hybrid}=\varepsilon \times GF_{hybrid}\times R_{hybrid}=700\times 10^{-6}\times 7.71\times 199.3\;\Omega =1.076\;\Omega$$

**Figure 10:**
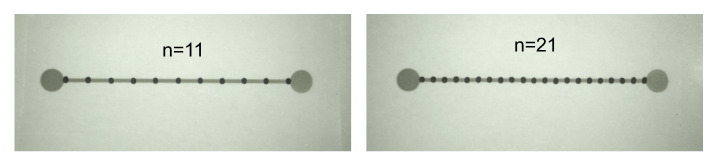
Strain sensors with eleven (n = 11) and twenty one (n = 21) sensing elements.

**Figure 11:**
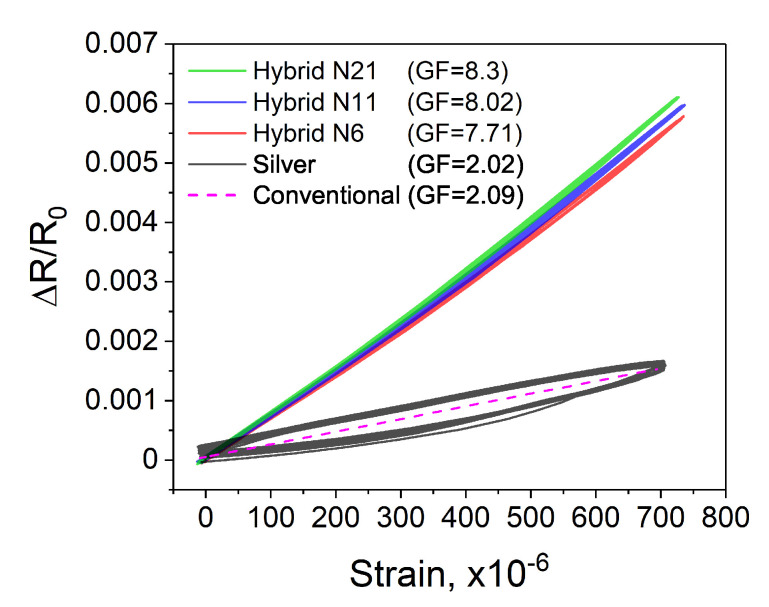
Calibration curves of the sensors with hybrid construction and the sensor entirely made of silver. For reference, the calibration curve of the conventional sensor was demonstrated.

The other important evaluation of the proposed sensor design was related to the impact of the number of resistive elements on strain sensitivity. Owing to the specific design of the sensor, each resistive element besides the intrinsic electrical resistance adds two contract resistances between the $R_{r}$ and $R_{c}$. The impact of the contact resistance changes on strain sensitivity was evaluated based on the comparative analysis of sensors with various number of the incorporated resistive elements. For this purpose, an additional two types of sensors were fabricated. The sensors with eleven (n = 11) and twenty one (n = 21) carbon droplets are demonstrated in Figure 10. The sensors were analyzed in the same manner as the previously described sensor incorporating n = 6 resistive elements. The collected data are shown in Figure 11. The calculated sensitivities for all sensors with hybrid construction were 7.71, 8.02, and 8.3 for n = 6, n = 11, and n = 21 of resistive elements, respectively. The observed effect of larger “n” values on the increase in GF is noticeable but not significant. On comparing the sensors with n = 6 and n = 21 of sensing elements, although the number of sensing elements and thus the number of contact resistances increased 3.5 times (or by 250%), the sensitivity of sensor increased only by 7.65%. The effect of the change in contact resistance on the strain sensitivity in the entire hybrid sensor structure is rather low. It appears that the intrinsic resistance changes in the carbon layer, i.e., piezoresistive effect is a dominant factor that defines the strain sensitivity of the demonstrated sensors.

Figure 11 shows that the silver sensor exhibits clearly noticeable hysteresis, which is however not seen in the sensors with the hybrid construction. The presence of hysteresis is often associated with the presence of residual binders between the particles of main filler material. Although the exact chemical composition of inks is kept confidential by the ink manufacturers, based on previous research, it can be stated that the residual binders may affect the mechanical properties of strain sensors and cause a viscoelastic effect at larger strains. A similar effect was reported by other researchers [32]. The carbon ink used in this study was already evaluated in our previous work [24]. It was demonstrated that the used carbon ink exhibits no hysteresis and linear output signal. In this study, the hysteresis was seen only in the silver sensor, which is however not seen in the sensors with the hybrid construction. It is a part of evidence wherein the electrical changes of the silver pattern have negligible impact on the strain sensitivity of sensors with the hybrid design that depends mainly on the properties of the functional resistive ink.

### 3.4. Temperature Sensitivity Analysis

**Figure 12:**
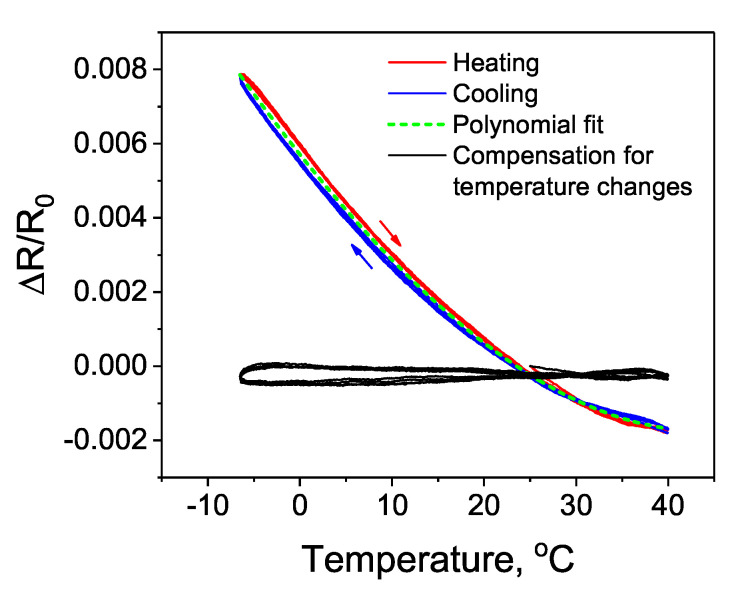
Temperature sensitivity of the developed strain sensor and an example of the compensation for temperature changes based on a reference temperature measurement.

In the last step, the developed sensor was analyzed in terms of sensitivity to temperature changes. During the analysis, the sensor was placed inside the environmental test chamber for continuous measurement of five cycles within the temperature range from −10 ${}^{\circ }$C to 40 ${}^{\circ }$C. The environmental test chamber was programmed to change the temperature at a constant rate of 1 ${}^{\circ }$C per min. During the measurement, the sensor was not subjected to any mechanical deformations. The results in Figure 12 reveal relatively high sensitivity to temperature changes that may affect strain measurements if performed at various temperatures.

This is a common problem related to most printed strain sensors that should not be omitted. Previous studies have also reported the same issue [4,32,33]. Although printed strain sensors are promising in terms of fabrication cost, scalability, and often high strain sensitivity, most of them suffer from relatively high sensitivity to temperature changes. One of the common practices to overcome this problem is the use of half- or full-Wheatstone-bridge circuit to compensate for temperature changes [23,24]. Another possible method that was implemented in this study relies on a reference temperature measurement. If the output signal from the sensor is reproducible during the changes in cyclic temperature, an equation describing its temperature characteristic can be found (Figure 12). In this study, the relationship between resistance change and temperature was described by the cubic polynomial function (Equation (5)). The coefficients A, B and C describe the trend line associated with the resistance change (*y*) due to temperature variations (*T*). The found values of A, B and C were 1.378 $\times \;10^{-8}$, 2.641 $\times \;10^{-6}$, −3.126 $\times \;10^{-4}$, respectively.

(5) $$y=\left(A\cdot T^{3}\right)+\left(B\cdot T^{2}\right)+\left(C\cdot T\right)$$

Next, based on the reference temperature measurement and the Equation (6) (that incorporates the Equation (5)), the change in the measured output signal due to temperature variations was corrected in real-time in a computer program, according to the following procedure. During the measurement, the output signal from the strain sensor ($m_{i}$) along with the temperature values was recorded in subsequent iterations (*i*) executed in the computer program. To calculate the corrected output signal (*Y*), from the measured data ($m_{i}$) the trend line described by the previously found Equation (5) was real-time subtracted in the consecutive iterations, depending on the measured temperature $T_{i}$. An offset to the y-intercept was done by subtracting $Y_{0}$, i.e., the first found $Y_{i}$ value (at the first iteration i=0). All the computations were performed automatically using the specially prepared computer program for data acquisition. The achieved results demonstrate a relatively stable output signal within the analyzed temperature range (Figure 11).

(6) $$Y_{i}=m_{i}-\left[\left(A\cdot T_{i}^{3}\right)+\left(B\cdot T_{i}^{2}\right)+\left(C\cdot T_{i}\right)\right]-Y_{0}$$

On the contrary, although it is possible to compensate for temperature changes, it might be inconvenient or technically difficult for many practical applications. It appears that there is a need for materials that exhibit low sensitivity to temperature changes. However, it is challenging to optimize the ink properties to match all requirements for printable strain sensors that include good sensing properties, low temperature sensitivity, good printability, and a reasonable cost. The fabrication process proposed herein is very flexible in terms of requirements for the printability. The functional ink can be deposed using a dispenser or an ordinary needle. Thus, the implementation of this simple technique may be very promising for the fast prototyping of strain sensors made of various composite materials. One of the materials that might be considered are CNT with combined doping of HNO${}_{3}$ and SOCl${}_{2}$ and with the addition of PEDOT:PSS capping layer [34]. It was demonstrated that such materials combination exhibit essentially no change in resistance upon heating up to 80 ${}^{\circ }$C and upon returning to room temperature. Another interesting material that can be potentially suitable to fabricate stable strain sensors is the CNT-CuI hybrid structure [35]. It was shown that such material exhibits stable electrical characteristics within a wide range of temperatures, which is a highly desirable feature for strain sensors.

## 4. Conclusions

In this study, the proposed sensor design was based on a hybrid construction composed of conductive intermittent pattern with embedded single droplets of resistive carbon-based ink. The key feature of the demonstrated sensor is that although the conductive pattern comprise the majority of the entire sensor area, the strain sensitivity depends almost selectively from the resistance changes in the small resistive elements. Although the volume resistivity of the carbon ink was 1.0 × 10${}^{-1}\;\Omega \;\cdot$ cm, the total electrical resistance of the sensor with six embedded resistive elements was only 199.3 ± 8.4 Ω. Such a segmented structure may be especially suitable for the manufacturing of sensors longer that those demonstrated herein and with a desirable resistance. For relatively long sensors, the electrical resistance is important with regard to power consumption (if the resistance is too low) and electrical noise level (if the resistance is too high). As the deposition of functional ink was limited to several single droplets, the amount of ink needed to build a sensor can be significantly reduced. It makes the sensors cost-effective and simple for fabrication. This feature can be especially promising for materials that exhibits good mechanical properties and are yet relatively expensive, similar to carbon nanotubes or graphene. The other advantage of the proposed sensor design is that diverse inks with a wide range of viscosities can be easily implemented to build the resistive elements of the sensor. When used, the dispenser the ink rheology does not require a strict optimization process in terms of printability. It might be very promising for fast prototyping of strain sensors made of various composite materials.

We would like to emphasize that this paper is not intended to undermine the functionality of other types of printed strain sensors entirely made of functional materials. In this study, we evaluate the alternative sensor design that might be considered with regard to its demonstrated features. Although further research related to the various construction of the intermittent structure and analysis of other resistive functional materials is in progress, the results demonstrated herein are very promising. All sensors exhibit good strain sensitivity and linear output signal within the analyzed strain range. Our current research focuses on the development of larger strain sensors, ranging from several to dozen of cm in length. Such sensors will be dedicated for human motion monitoring and structural health monitoring where larger sensing systems are desirable.
